# Functional Nanoporous Materials

**DOI:** 10.3390/nano10040699

**Published:** 2020-04-07

**Authors:** Christian Weinberger, Michael Tiemann

**Affiliations:** Department of Chemistry, Paderborn University, Warburger Str. 100, D-33098 Paderborn, Germany

This Special Issue on “Functional Nanoporous Materials” in the MDPI journal nanomaterials features seven original papers. Six of them deal with experimental work, and one of them investigates nanoporous materials from a theoretical point of view. They cover a wide range of materials, starting from porous organic polymers over silicon and silver to metal oxides and silica. A wide range of applications, such as gas adsorption and separation, sensing, solar cells, and catalysis are discussed.

Xu et al. [1] utilize a Sonogashira coupling of 1,3,5-triethynylbenzene with terephthaloyl chloride to form a novel ynone-linked microporous organic polymer (y-POP). Postsynthetic functionalization of y-POP with tris(2-aminoethyl) amine (tren) enables access to amino-functionalized polymers. The pristine material reaches a surface area up to 230 m^2^ g^−1^. Increasing the number of amino groups within the material reduces the surface area. The authors propose that this is due to the pore-blocking effect of the tren species. The highest amine loading is 19%, which reduces the surface area derived by Brunauer–Emmett–Teller method [2] (BET surface area) to 85 m^2^ g^−1^. Due to the microporous structure and the high amount of amine species, the authors investigate the CO_2_ adsorption capacity and CO_2_-over-N_2_ selectivity of the pristine and functionalized y-POP materials. Experimental data prove that the amine loaded samples exhibit a higher CO_2_ capacity and preferential adsorption of CO_2_ in the presence of N_2_.

Ivanada, Gamulin, and coworkers [3] exploit silver-coated porous photonic crystals as surface-enhanced Raman scattering (SERS) substrates utilizing a near-infrared excitation wavelength of 1064 nm. Its considerable penetration depth into silicon causes photoluminescence, which conceals with the SERS signal with a broad photoluminescence peak. Thus, a porous photonic crystal is used to quench the photoluminescence of the crystalline silicon. The SERS activity was investigated in an aqueous/ethanolic solution of two dyes, namely, rhodamine 6 G (R6G) and crystal violet (CV). The investigators show that the detection limit of the dyes is 10^−7^ M (R6G) and 5·10^−8^ M (CV), respectively. These concentrations are about five orders of magnitude lower compared to bare porous silicon.

Zhang and Zhang [4] investigate the free vibration and buckling of functionally graded (FG) nanoporous metal foam (NPMF) nanoshells. The authors are using the established first-order shear deformation (FSD) shell theory and Mindlin’s (most general) strain gradient theory. With regards to the structural composition, symmetric and unsymmetric nanoporosity distributions are taken into account. The study analyses the effect of a nanoporosity coefficient, as well as the length scale and geometrical parameters on the mechanical behavior of FG NPMF nanoshells.

A study from Giraldo and his team [5] describes the thermal decomposition of potassium permanganate at 400 °C and 800 °C. As a function of the temperature, the composition of the decomposition product varies between triclinic $K_{0.29}^{+}\left({\mathrm{Mn}}_{0.84}^{4+}\;{\mathrm{Mn}}_{0.16}^{3+}\right)O_{2.07}\cdot 0.61\;H_{2}O$ and hexagonal $K_{0.48}^{+}\left({\mathrm{Mn}}_{0.64}^{4+}\;{\mathrm{Mn}}_{0.36}^{3+}\right)O_{2.06}\cdot 0.50\;H_{2}O$, respectively. The materials exhibit a BET surface area between 5-16 m^2^ g^−1^ with a broad pore size distribution. The authors suggest that their synthesized materials might be utilized as a catalyst, and therefore they studied the charge transport mechanism by electrical impedance analysis. The crystallite size, manganese’s average oxidation state, and the crystal symmetry influence the impedance measurements. Both materials exhibit semiconducting properties and thermally activated electron “hopping”. 

Smått et al. [6] investigate how well-defined pinholes in TiO_2_ electron selective layers (ESL) in planar heterojunction perovskite solar cells influence the device performance. Defects such as pinholes compromise the cell performance due to enhanced surface recombination of electron-hole pairs. Sol-gel derived porous titania layers were exploited as ESL, synthesized in a dip coating process, in which block copolymers were utilized as templates. The porosity of TiO_2_ was varied between 0% and 47%, as well as the film thickness between 20 and 75 nm. It turns out that narrow pinholes (<10 nm) do not affect the device performance, which might be attributed to the fact that the perovskite crystals do not form a connecting path through the pores in the titania layer up to the electrode. Thin titania layers (<20 nm), lead to incomplete surface coverage. Hence a drop in performance of the device can be observed. The scientists around Smått present an ideal model system to investigate the effect of pinholes on the solar cell performance, leading to efficiency values up to 14.1%.

Björk and her colleagues [7] show how ordered mesoporous SBA-15 silica particles grow on surfaces. The particle-based approach to synthesize silica films that can be functionalized and used as catalysts for esterification reactions leads to a film thickness between 80 and 750 nm. It can be tuned by the addition of NH_4_F during synthesis because it influences the formation rate of silica particles. The time of the addition into the mixture is crucial for the quality of the resulting film. Under optimal conditions, the homogenous surface coverage of an area larger than 75 cm^2^ is possible and independent of the shape of the substrate (flat or three dimensional). Furthermore, they present surface functionalization with acetic acid through co-condensation and a post-synthetic coating with furfuryl alcohol foams. In the latter case, the alcohol can be converted to carbon. A second surface functionalization leads to a sulfonated CMK-5 carbon-SBA-15 silica composite material. The carbon-coated films were used as a catalyst for the esterification between acetic acid and ethanol, reaching conversion up to 30% within one hour compared to a 5% conversion rate compared to the catalyst-free reaction.

Another study dealing with ordered mesoporous silica materials is presented by Wilhelm et al [8]. They show how to functionalize porous silica materials with various phosphonic acids, phosphonic acid esters as well as adenosine monophosphate. A wide range of silica materials were investigated to cover a broad range of surface areas and pore sizes, e.g., MCM-41 with a pore size around 4 nm and a BET surface area of around 1300 m^2^ g^−1^. Furthermore, ordered mesoporous SBA-15 silica (pore size 6 nm, surface area 630 m^2^ g^−1^) was utilized. Additionally, commercially available LiChrosorb SI 100 (14 nm, 280 m^2^ g^−1^) and synthesized disordered silica with hierarchical meso- and macro-porosity were exploited. Lichrosorb and SBA-15 silica samples with immobilized (4R)-4-phosphonooxy-l-proline were investigated in terms of their catalytic potential in the conversion cyclohexanone with 4-nitrobenzaldehyde in an asymmetric aldol reaction.

This collection of fine articles shows the potential and challenges in the characterization and application of functional nanoporous materials. Both from a theoretical and experimental point of view, the authors present novel ideas, showing the prospects and exciting developments in the field of porous materials.
